# Antimicrobial Photodynamic Therapy With New Methylene Blue and Its Combined Effect With Antibiotics Against MDR *Acinetobacter baumannii*

**DOI:** 10.1002/mbo3.70342

**Published:** 2026-06-23

**Authors:** Barat Barati, Tahereh Navidifar, Mohsen Ostovari, Roya Ghanavati, Atieh Darbandi

**Affiliations:** ^1^ Department of Radiology Technology Shoushtar Faculty of Medical Sciences Shoushtar Iran; ^2^ Department of Basic Sciences Shoushtar Faculty of Medical Sciences Shoushtar Iran; ^3^ Department of Medical Physics and Medical Engineering, School of Medicine Shiraz University of Medical Sciences Shiraz Iran; ^4^ Ionizing and Non‐ionizing Radiation Protection Research Center (INIRPRC) Shiraz University of Medical Sciences Shiraz Iran; ^5^ Department of Basic Sciences Behbahan Faculty of Medical Sciences Behbahan Iran; ^6^ Molecular Microbiology Research Center Shahed University Tehran Iran

**Keywords:** Antimicrobial Photodynamic Therapy, Colistin, MDR Acinetobacter baumannii, New Methylene Blue, Tigecycline

## Abstract

The present study aimed to evaluate the antibacterial efficacy of new methylene blue‐mediated antimicrobial photodynamic therapy (NMB‐mediated aPDT) in combination with tigecycline and colistin against multidrug‐resistant (MDR) *A. baumannii* and to explore ROS production as a possible mechanism contributing to the enhanced antibacterial activity of the combined treatments. MIC determination, sublethal irradiation and aPDT assays, checkerboard analysis, and ROS detection were performed. MIC of NMB for this MDR strain was greater than 100 μg/mL. Under various radiation times, aPDT at 12.5 μg/mL NMB was considered a sublethal dose that represented with a reduction of 1.7–2.6 log_10_ compared with control, and aPDT at 25 and 50 μg/mL were considered lethal doses that resulting in more reduction of bacterial counts to 3.23–4.3 log_10_ CFU/mL and 2.92–4.0 log_10_ CFU/mL, respectively. The irradiated NMB at 25 μg/mL and tigecycline at 1/4 × MIC showed a synergistic interaction. In addition, multiple additive interactions were observed at other concentrations of NMB and tigecycline, as well as with colistin at sub‐MIC concentrations, and these findings were further confirmed by CFU counting. The increased production of ROS was effectively detected following aPDT alone and in combination of antibiotic with aPDT. These findings suggest that NMB‐mediated aPDT alone and in combination with colistin or tigecyclines can be a promising approach to eradicate infections associated with MDR *A. baumannii* and reduces the development of further antibiotic resistance.

## Graphical abstract

NMB‐mediated aPDT significantly inhibited MDR *A. baumannii* and enhanced the efficacy of tigecycline and colistin through synergistic or additive interactions. Increased ROS generation during aPDT and combination therapy may contribute to the observed antibacterial effects.

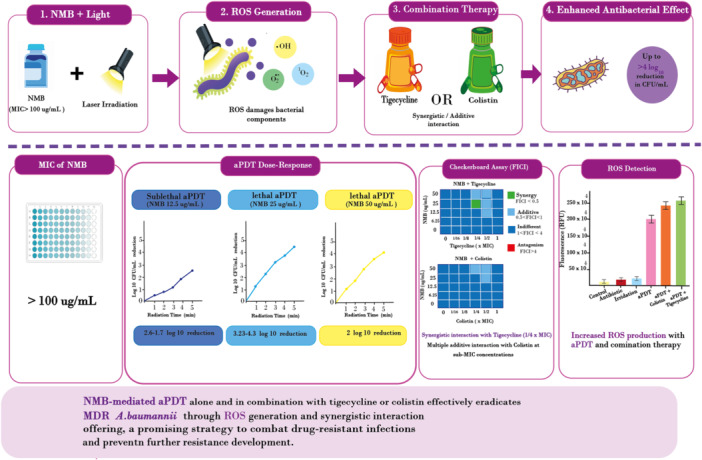

## 1 Introduction

*Acinetobacter baumannii* is a significant opportunistic pathogen that is responsible for healthcare‐associated infections and has been implicated in several hospital‐acquired infections, including sepsis, endocarditis, pneumonia, meningitis, and wound infections. Some risk factors predisposing patients to hospital‐acquired infections caused by *A. baumannii* are immunocompromised status, long hospital stay, invasive medical procedures, prior broad‐spectrum antibiotic exposure, and admissions to the Intensive Care Unit (ICU) (Antunes et al. 2014). Multidrug‐resistant (MDR) and extensively drug‐resistant (XDR) clones of *A. baumannii* are emerging in hospital‐acquired infection settings. However, there are limited antibiotic therapies for their eradication; hence, there is an urgent need for the introduction of new therapeutic options (Ayoub Moubareck and Hammoudi Halat 2020).

In addition, the ability of *A. baumannii* for biofilm formation can enhance its transmissibility within the hospital setting and is a major factor in the emergence of nosocomial infections, particularly ventilator‐associated pneumonia and catheter‐associated infections (Gedefie et al. 2021). Therefore, the development of new therapeutic approaches for the effective treatment of this bacterium is necessary.

One of the alternative and promising strategies for the treatment of MDR *A. baumannii‐*associated infections is antimicrobial photodynamic therapy (aPDT). Two key components in PDT are a non‐toxic photosensitizer (PS) and visible or ultraviolet light for the generation of cytotoxic reactive oxygen species (ROS) (Li et al. 2024). PSs have a static electronic structure with a ground state. When irradiated with light of a specific wavelength, the PS is excited from its ground state to an excited state. In addition, aPDT exerts its toxic effects on microbes through a multi‐target mechanism, unlike the single‐target mechanism caused by conventional antibiotics. Since aPDT affects more than a single molecular target, it may reduce the possibility of emerging bacterial resistance (Hu et al. 2018).

Phenothiazinium dyes represent a class of first‐generation synthetic PSs that were initially explored as anticancer PDT agents. However, due to their cationic nature, these compounds possess a high affinity for Gram‐positive and Gram‐negative bacteria and, therefore, they are the most widely used in aPDT in clinical practice. In fact, phenothiazine derivatives such as methylene blue (MB) and toluidine blue O (TBO) have been the most widely used PSs to date (Ghorbani et al. 2018). MB has received regulatory approval for usage in aPDT of dental infections (Vecchio et al. 2015). However, its activity is comparatively limited, and most research laboratories have attempted to synthesize new compounds with improved photodynamic activity (Hamblin 2016).

A few of these novel PSs, such as dimethyl methylene blue (DMMB) and new methylene blue (NMB), which are derivatives of MB, contain a much higher cationic charge with a more lipophilic nature, rendering them more active against bacterial cells (Gollmer et al. 2015). Ragas et al. investigated four phenothiazinium dyes, including TBO, MB, DMMB, and NMB, for aPDT against MDR *A. baumannii* and confirmed that NMB was the most effective PS, achieving a 3.2‐log reduction in bacterial growth (Ragàs et al. 2010).

To the best of our knowledge, no previous study has specifically investigated the combined antibacterial interaction between NMB‐mediated aPDT and last‐resort antibiotics against MDR *A. baumannii*. Moreover, the potential role of ROS generation during combined NMB‐aPDT‐antibiotic treatment has not yet been clarified. Therefore, the present study was aimed to evaluate the antibacterial efficacy of NMB‐mediated aPDT in combination with tigecycline and colistin and further explored ROS production as a possible mechanism contributing to the enhanced antibacterial activity of the combined treatments.

## 2 Materials and Method

### 2.1 Strains and Culture Conditions

We obtained an MDR, weak biofilm‐producing *A. baumannii* AB05 strain from a previous study; this clinical isolate was recovered from the urine sample of a burn patient admitted to Taleghani Burn Hospital in Ahvaz, Iran. The isolate exhibited high‐level resistance to cefepime (MIC: 512 μg/mL), amikacin (MIC: 256 μg/mL), and meropenem (MIC: 256 μg/mL), while the MIC values for colistin and tigecycline were 1 μg/mL and 32 μg/mL, respectively (Amin et al. 2019). *A. baumannii* was cultured in tryptic soy broth (TSB) (Merck, Germany) aerobically at 37°C overnight. The bacterial suspension was diluted to a final concentration of 1.0 × 10^6^ colony‐forming units (CFU)/mL, with an optical density at 600 nm (OD_600_) of 0.02.

The stock solution of NMB (Sigma‐Aldrich, Steinheim, Germany) was prepared at a concentration of 0.4 mg/mL in distilled water, sterilized by filtration through a 0.22‐μm filter, and stored at 4°C in the dark until use. The diode laser (DL) (Konftec, Taiwan and A.R.C. Laser GmbH, Nuremberg, Germany) had a wavelength of 632 nm and an output of 200 mW for photodynamic activation. The laser output was controlled with a power meter (Laser Point s.r.l., Milan, Italy).

### 2.2 Determination of NMB Minimum Inhibitory Concentration (MIC)

The broth microdilution assay was conducted to determine the MIC of NMB for the MDR *A. baumannii* strain. Briefly, 100 μL of TSB was placed in each well of a 96‐well microtiter plate. Next, 100 μL of the NMB stock solution (0.4 mg/mL) was added to the first well of each row (column 1), and serially diluted twofold from columns 2 to 10. Subsequently, 100 μL of bacterial suspension (5.0 × 10^6^ CFU/mL) was added to each well so that the final bacterial concentration was 2.5 × 10^6^ CFU/mL per well. The range of NMB concentrations in the wells was 100 to 0.19 μg/mL. Column 11 served as the positive growth control (bacterial suspension without NMB), and column 12 served as a negative control (medium and NMB, without bacteria). The plates were then incubated overnight at 37°C in the dark. The MIC was defined as the lowest concentration that completely inhibited visible bacterial growth. For the colony counting, a 10 μL aliquot from each well was serially diluted in PBS, plated on TSA, incubated overnight at 37°C and CFUs were counted. Colony counting was performed as a complementary viability assessment and was not used for formal MBC determination. Each experiment was performed in triplicate (Pourhajibagher et al. 2016a; Pourhajibagher et al. 2016b).

### 2.3 Determination of the Sublethal Diode Laser (sDL) Irradiation Time

First, 200 μL of the adjusted bacterial suspension with a final concentration of 5.0 × 10^6^ CFU/mL was introduced into each well of the 96‐well microtiter plate. The plate was then positioned at a consistent distance of 4 cm from the illumination head of the laser and subjected to DL irradiation at a wavelength of 632 nm and ambient temperature for time periods of 1, 2, 3, 4, and 5 min. The fluence values at these exposure times were calculated to be 23.43, 46.87, 70.31, 93.75, and 117.18 J/cm^2^, respectively. Control samples were treated similarly but without laser irradiation. Black paper was placed under the microplate to minimize light scattering and reflection during irradiation (Pourhajibagher et al. 2016a; Pourhajibagher et al. 2016b). The diameter of the irradiation area was equivalent to the diameter of the well base (6.39 mm). Sublethal doses (sLDs) of irradiation were determined as a 0.5–2 log_10_ reduction in CFU/mL, as the changes in survival rate of treated bacteria compared with untreated bacteria (Wozniak et al. 2019).

### 2.4 Determination of Sublethal Dose of aPDT

To determine sublethal photodynamic doses (sPDTs), first 96‐well microtiter plate was filled with 100 μL of TSB. Then, 100 μL of NMB at 2 × MIC was serially diluted two‐folds to 1/8 × MIC. Finally, 100 μL of bacterial suspension with a concentration of 5.0 × 10^6^ CFU/mL was inoculated into each well. After inoculation, the concentration of NMB was in the range of 1/2 to 1/32 × MIC from column 1 to column 5. The microplates were incubated for 5 min in the dark and subjected to laser irradiation at a wavelength of 632 nm and ambient temperature for time periods of 3, 4, and 5 min, represented to fluence values 70.31, 93.75, and 117.18 J/cm^2^, respectively (Pourhajibagher et al. 2016b). The control group did not have any treatment, and all experiments were performed in triplicate. In addition, dark‐control and light‐control experiments were performed independently to evaluate the effects of NMB alone and diode laser irradiation alone, respectively. The antibacterial activity observed following aPDT was interpreted by comparison with both untreated bacteria and these single‐treatment controls to distinguish photodynamic effects from the individual effects of NMB or light exposure.

The sPDTs were defined as a 0.5–2 log_10_ reduction in CFU/mL as the changes in survival rate of treated bacteria compared to untreated bacteria. Also, a reduction of 3 or more log_10_ in CFU/mL as the changes in survival rate of treated bacteria compared to untreated bacteria was defined as the lethal dose. The colony counting was conducted as in the previous sections (Wozniak et al. 2019).

### 2.5 Checkboard Assay

Checkerboard microdilution assay was performed to evaluate the interaction between NMB‐mediated aPDT and antibiotics. Briefly, 100 μL of TSB was added to each well of a sterile 96‐well microtiter plate. Two‐fold serial dilutions of 2× working solutions of tigecycline or colistin were prepared horizontally across the rows (A to F), while two‐fold serial dilutions of 2× NMB working solutions were prepared vertically across the columns (1 to 6). The use of 2× working solutions was intended to compensate for the subsequent dilution caused by the addition of the bacterial inoculum.

Subsequently, 100 μL of bacterial suspension adjusted to 5.0 × 10^6^ CFU/mL was added to each well to achieve the final assay volume. Following inoculation, the final concentrations of both antibiotics and NMB in the wells corresponded to the sub‐MIC concentrations of 1/2×, 1/4×, and 1/8× MIC.

The plates were incubated in the dark for 30 min to allow NMB uptake and then exposed to diode laser irradiation at 632 nm with a fluence of 70.3 J/cm^2^. Following irradiation, the plates were incubated at 37°C for 16 h, and optical density was measured at 580 nm.

To further evaluate bacterial survival, 10 μL aliquots from each well were serially diluted, plated onto tryptic soy agar (TSA), and incubated overnight at 37°C for CFU enumeration (Wozniak et al. 2019). Wells containing bacteria without antimicrobial agents or light exposure served as positive growth controls, while wells containing sterile medium and NMB without bacterial inoculation served as negative controls to confirm sterility and baseline absorbance.

The interaction between aPDT and the antibiotics was determined by the fractional inhibitory concentration index (FICI). The FIC of each drug (FICA and FICB) was calculated as the MIC of the drug in combination divided by the MIC of the individual drug. The overall FICI was calculated using the formula: ΣFICI = FICA + FICB. According to traditional interpretive criteria, the interaction is synergistic if FICI ≤ 0.5, additive if 0.5 < FICI ≤ 1.0, indifferent if 1.0 < FICI < 4.0, and antagonistic if FICI ≥ 4.0 (Wozniak et al. 2019).

### 2.6 ROS Detection

A suspension of *A. baumannii* of approximately 1 × 10^8^ cells/mL was prepared in 1× phosphate‐buffered saline (PBS) and centrifuged at 6500 rpm for 5 min to pellet the cells. The cells were resuspended in 100 μL of a solution containing 5 μM 3′‐p‐aminophenyl fluorescein (APF) in PBS. The cell suspension was incubated in the dark for 30 min at 35°C to allow the uptake of the ROS‐sensitive probe.

Following incubation, cells were centrifuged and washed to remove excess APF before being resuspended in 100 μL of one of the treatment conditions described below: PBS (negative control), antibiotic alone, NMB alone, or both antibiotic and NMB. Following a 5‐min dark incubation period to allow interaction, suspensions were exposed to an illumination dose of 70.3 J/cm^2^ (200 mW/cm^2^ for 3 min). The samples were then transferred to a black 96‐well plate, and fluorescence was measured in a plate reader at excitation/emission wavelengths of 490/515 nm. Blanks for the control samples without APF were used, and their fluorescence values were subtracted to normalize the APF‐treated samples (de Freitas et al. 2018).

### 2.7 Statistical Analysis

In this study, all experiments were replicated three times, and the results are presented as mean ± standard deviation (SD). Prior to analysis, viable bacterial counts (CFU/mL) were log10‐transformed. The normality of the transformed data was assessed using the Shapiro–Wilk test. Since the data showed no significant deviation from a normal distribution, statistical comparisons between treatment groups and the untreated control were performed using one‐way analysis of variance (ANOVA) followed by Dunnett's multiple comparisons test. Statistical analyses were done using GraphPad Prism (GraphPad Prism Software, USA). A *p*‐value < 0.05 was considered to be statistically significant.

## 3 Results

### 3.1 Inhibition of MDR A. baumanni Growth By NMB

**Figure 1:**
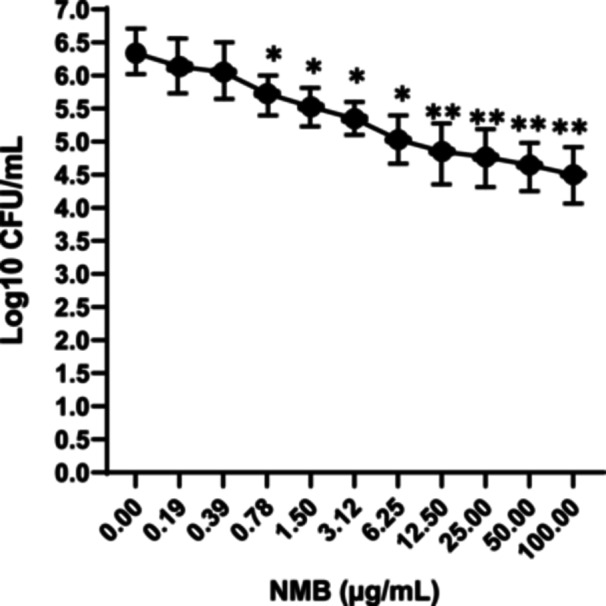
MIC of NMB, *p < 0.05 and **p < 0.01.

The antibacterial activity of NMB against the MDR *A. baumannii* strain was evaluated using the broth microdilution method. As shown in Figure 1, the bacterial viability, based on log10 CFU/mL, gradually decreased with increasing concentrations of NMB in a dose‐dependent manner. A statistically significant decrease in viable counts was observed at concentrations of 0.78 μg/mL and above (*p* < 0.05). Moreover, the unexposed group showed an approximate mean of 6.4 log10 CFU/mL, whereas treatment groups with high NMB concentrations (≥ 12.5 μg/mL) indicated a clear decrease in the viable bacterial counts to around 4.85 log10 CFU/mL and lower, corresponding to a mean reduction of 1.55 log10 CFU/mL and lower compared with the control group. No concentration within the tested range (0.19–100 μg/mL) completely inhibited visible bacterial growth, indicating that the MIC of NMB for this MDR strain was greater than 100 μg/mL.

### 3.2 Inhibition of MDR A. Baumanni Growth By Sublethal Diode Laser (sLD) Irradiation Time

**Figure 2:**
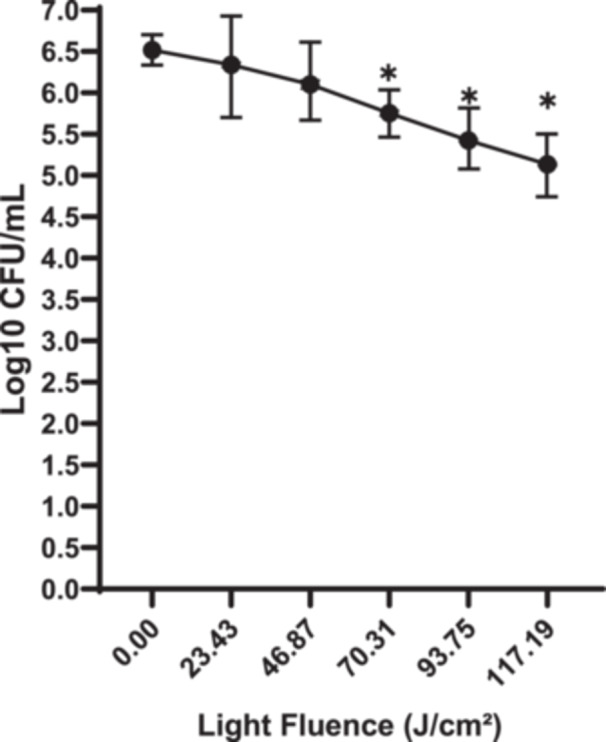
Effect of Diode Laser Fluence on MDR *A. baumannii* Viability *p < 0.05.

The effect of DL irradiation on the viable bacterial count of the MDR *A. baumannii* strain was evaluated at various fluences ranging from 23.43 to 117.18 J/cm^2^. As shown in Figure 2, the viable bacterial counts exhibited a gradual decrease with increasing energy density in a fluence‐dependent manner. The unexposed group exhibited an approximate mean of 6.4 log_10_ CFU/mL, whereas exposure to high fluence values (≥ 70.31 J/cm^2^) reduced the viable bacterial counts to around 5.74 log_10_ CFU/mL and lower. The fluences of 70.31 to 117.18 J/cm^2^ produced a significant reduction of approximately 0.75 to 1.4 log10 CFU/mL compared with the unexposed group (*p* < 0.05) and were defined as sLDs.

### 3.3 Antibacterial Activity of NMB‐Mediated Apdt Against MDR A. baumannii

**Figure 3:**
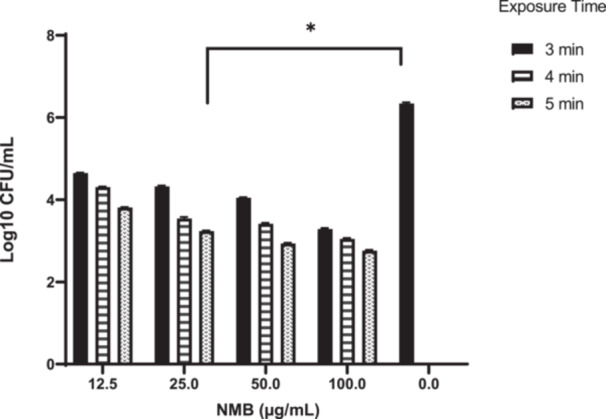
NMB‐mediated aPDT at concentrations of 12.5–100 µg/mL under three fluence values of 70.31, 93.75, and 117.18 J/cm^2^. All aPDT‐treated groups showed significantly lower viable counts than the untreated control group (0 µg/mL NMB), as indicated by the asterisk (*). *p* < 0.05 compared with the untreated control group.

The antibacterial activity of NMB‐mediated aPDT against the MDR *A. baumannii* strain was evaluated at four sub‐MIC concentrations (12.5 to 100 μg/mL) of NMB combined with DL irradiation. As demonstrated in Figure 3, aPDT at all tested NMB concentrations under various fluence levels (70.31, 93.75, and 117.18 J/cm^2^) reduced the bacterial viability compared with the untreated sample in a concentration‐dependent manner.

At 12.5 μg/mL of NMB, aPDT reduced bacterial viable counts to 3.8–4.6 log_10_ CFU/mL, depending on the irradiation time, which represented a reduction of 1.7–2.6 log_10_ compared with the untreated sample. The higher NMB concentrations, 25 and 50 μg/mL, enhanced PDT effects, resulting in greater reduction of bacterial counts to 3.23–4.3 log_10_ CFU/mL and 2.92–4.0 log_10_ CFU/mL, respectively. The greatest PDT‐mediated reduction was found at 100 μg/mL with a reduction in bacterial count to 2.74–3.29 log_10_ CFU/mL, which represented a reduction of 3.0–3.6 log_10_ compared with the untreated group. Therefore, under various radiation levels, aPDT at 12.5 μg/mL NMB was considered a sublethal dose, and aPDT at 25 and 50 μg/mL were considered lethal doses. These findings indicate that the antibacterial activity resulted predominantly from the combined effect between NMB photoactivation and laser irradiation rather than from the independent effects of either treatment alone.

### 3.4 Synergistic Interaction Between NMB‐Mediated aPDT and Antibiotics Against MDR A. baumannii

**Figure 4:**
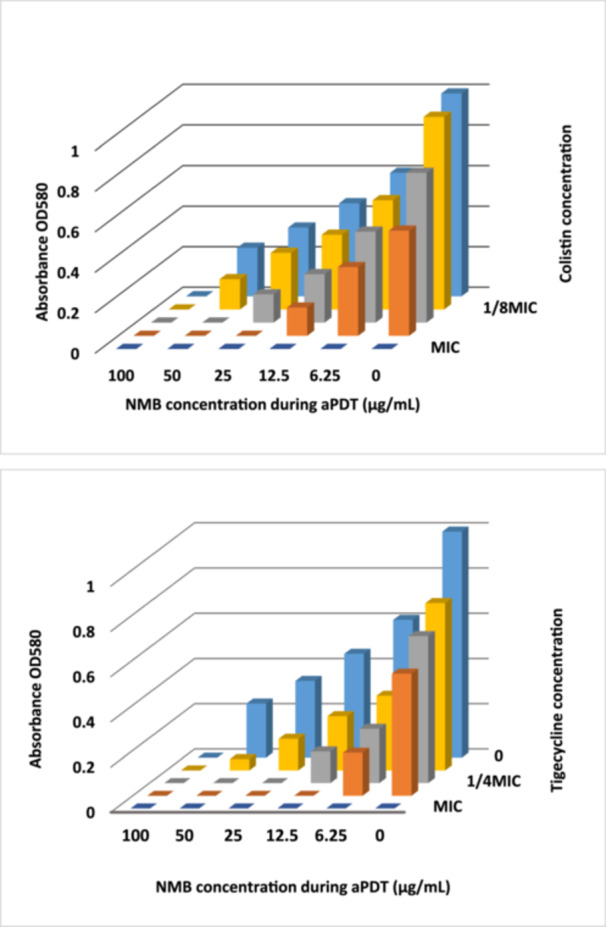
Checkerboard analysis of NMB‐mediated aPDT at a fixed fluence of 70.3 J/cm^2^ combined with sub‐MIC concentrations of tigecycline or colistin.

To evaluate synergistic interactions between serial dilutions of NMB at a fluence of 70.3 J/cm^2^ and tigecycline or colistin at sub‐MIC concentrations, the checkerboard assay was employed. As demonstrated in Figure 4, irradiated NMB at 25 μg/mL and tigecycline at 1/4 × MIC showed a synergistic interaction (FICI = 0.5). In addition, four additive interactions were observed, including irradiated NMB at 50 μg/mL and tigecycline at 1/4 × MIC (FICI = 0.75), irradiated NMB at 50 μg/mL and tigecycline at 1/2 × MIC (FICI) = 1, NMB at 25 μg/mL under irradiation and tigecycline at 1/2 × MIC (FICI = 0.75), and NMB at 12.5 μg/mL under irradiation and tigecycline at 1/2 × MIC (FICI = 0.625).

On the other hand, three additive interactions were observed between irradiated NMB at 25 and 50 μg/mL and colistin at several sub‐MIC concentrations. These include NMB at 50 μg/mL under irradiation and colistin at 1/4 × MIC (FICI = 0.75), NMB at 50 μg/mL under irradiation and colistin at 1/2 × MIC (FICI = 1), and NMB at 25 μg/mL under irradiation and colistin at 1/2 × MIC (FICI = 0.75).

**Figure 5:**
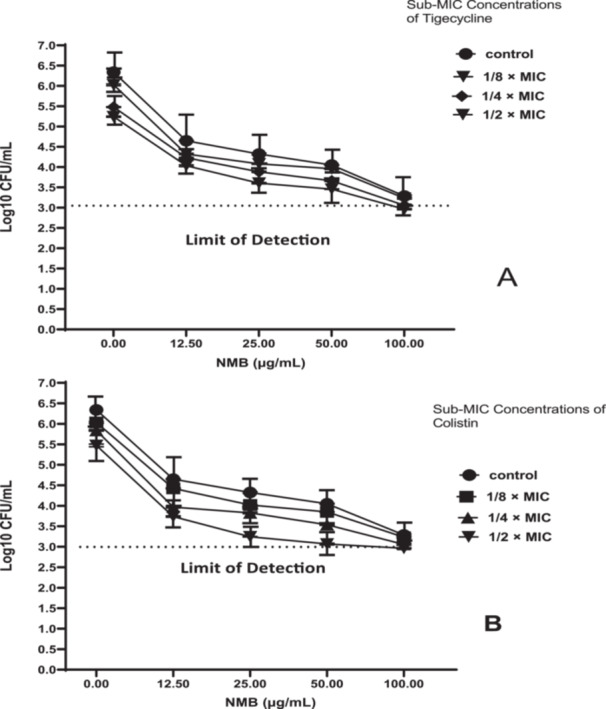
CFU counting in the presence of PDT and sub‐MIC concentrations of (A) tigecycline and (B) colistin.

**Figure 6:**
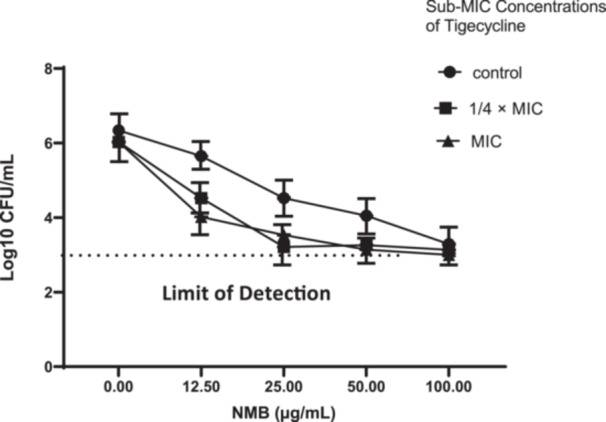
The synergic interaction between irradiated NMB at 25 μg/mL and tigecycline at 1/4 × MIC using colony counting.

Along with checkerboard analysis, CFU counting following PDT treatment and sub‐MIC concentrations of colistin and tigecycline was performed and confirmed the results of checkerboard analysis (Figure 5). Also, the synergistic interaction between irradiated NMB at 25 μg/mL and tigecycline at 1/4 × MIC using colony counting was demonestrated in Figure 6. Overall, the obtained results indicated that combined treatments led to more effective eradication of the MDR *A. baumannii* isolate compared with PDT alone.

### 3.5 ROS Generation in the Presence of aPDT and Combination Treatments

**Figure 7:**
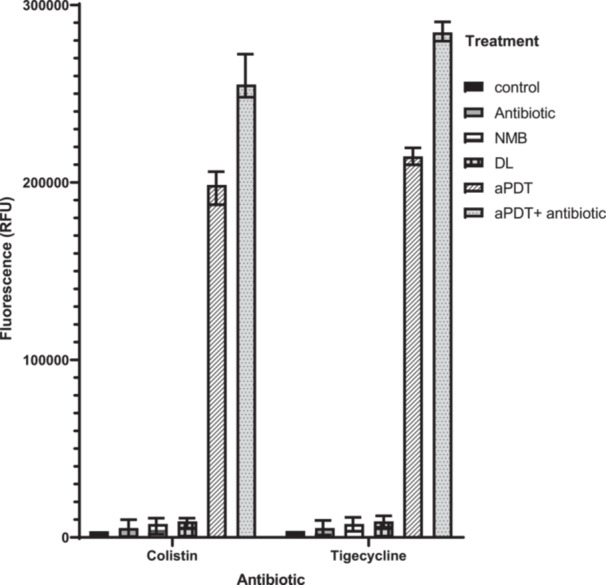
ROS generation in the presence of aPDT and combination treatments.

To evaluate whether ROS production is one of the possible mechanisms responsible for bacterial eradication by aPDT alone and in combination with antibiotics, the level of ROS (OH radicals) generated under various treatments was measured using 3′‐p‐aminophenyl fluorescein. As shown in Figure 7, treatment with antibiotic alone, NMB alone, or DL alone produced a few fluorescence signals. In contrast, the production of ROS was significantly increased upon treatment with aPDT alone and in combination with antibiotics.

Among the tested combinations, tigecycline‐mediated aPDT exhibited the greatest fluorescence value, approximately 284617 RFU, whereas the colistin‐mediated aPDT combination was approximately 255280 RFU. In contrast, the fluorescence values for antibiotic alone, NMB alone, and DL alone ranged from 5365 to 9034 RFU.

## 4 Discussion

Currently, PDT has mainly been used as a therapeutic option for the management of non‐invasive tumors, but is not yet applied routinely for the treatment of infections. Researchers are exploring the potential of PDT as an alternative approach for the eradication of bacterial infections due to its effectiveness against MDR microorganisms and low toxicity to normal cells in vivo (Cieplik et al. 2018). The primary action mechanism of PDT relies on the generation of ROS following the exposure of PS to irradiation. ROS production is associated with a wide‐ranging antibacterial activity, including disruption of some metabolic pathways and cellular structures rather than targeting of a single process or structure (Correia et al. 2021). Hence, aPDT can reduce the emergence of bacterial resistance. Another advantage of aPDT is its compatibility with other therapy strategies, such as antibiotics, peptides, and photothermal therapy (Naskar and Kim 2023).

In accordance with the proposed mechanisms above, our findings first indicated a significant reduction in bacterial count by NMB‐mediated aPDT at all examined concentrations of this PS after irradiation with several fluences (70.31, 93.75, and 117.18 J/cm^2^). These observations demonstrate an antibacterial activity dependent on fluence, and imply that adequate light exposure is essential for ROS‐dependent bacterial killing. Moreover, NMB‐mediated aPDT at 100 μg/mL under various irradiation doses reduced the bacterial count by 3.0–3.6 log_10_ compared with the untreated group. Also, the comparison with the corresponding dark and light‐only controls further confirmed that the observed bacterial inactivation was due to true photodynamic activity. In other words, this observation supports the idea that the antibacterial effects of NMB‐mediated aPDT were mainly due to the combined action of NMB and irradiation, instead of separate effects of NMB or irradiation alone. Consistent with the findings of our study, a few studies have investigated NMB‐mediated aPDT against pathogenic bacteria and fungi and confirmed its significant antibacterial activity (Vilela et al. 2025; Zheng et al. 2023; López‐Chicón et al. 2016). Moreover, Vilela et al. in Brazil found that NMB at 12.5 or 25 μM, after exposure to light for 15 and 30 min, inhibited the growth of non‐typhoidal *Salmonella enterica* serovars (Vilela et al. 2025). Also, Zheng et al. in China indicated that the efficiency of NMB‐aPDT against *Fonsecaea nubica* improved with higher light intensity, such that *F. nubica* was completely eradicated at 25 µmol/L NMB under a light dose of 40 J/cm^2^ and at 50 µmol/L NMB under light doses more than 30 J/cm^2^ (Zheng et al. 2023). López‐Chicón et al. in Spain also demonstrated that NMB at 50 μM fully killed all *Trichophyton mentagrophytes* cells under a 10‐min exposure at a fluence of 81 J/cm^2^. In addition, the increase in NMB levels to 100 μM reduced the required fluence to 9 J/cm^2^ (López‐Chicón et al. 2016). Overall, these findings suggest that the increase in NMB concentration may decrease the light fluence needed for effective microbial inhibition. This underscores the necessity of adjusting both PS concentration and irradiation intensity to enhance the antimicrobial efficacy of NMB‐mediated aPDT.

In the second phase, our study's findings confirmed the combined therapeutic effect of NMB‐mediated aPDT with sub‐MIC concentrations of the two last‐resort antibiotics, tigecycline and colistin, on the growth inhibition of *A. baumannii*. According to the results, we found a synergistic interaction between irradiated NMB at 25 μg/mL and tigecycline at 1/4 × MIC with FICI = 0.5, while multiple additive interactions were found at other concentrations of NMB and tigecycline, as well as with colistin at sub‐MIC concentrations, which were further confirmed by CFU counting. These results suggest that combining sublethal doses of NMB‐mediated aPDT with sub‐MIC concentrations of tigecycline or colistin can improve the antimicrobial efficacy against MDR *A. baumannii*. Similar to the results of our study, Ramírez et al. evaluated the interaction between endogenous PSs or methylene blue‐mediated aPDT and sub‐MIC concentrations of tigecycline against several Gram‐negative bacteria. However, in contrast to our results, they demonstrated antagonistic interactions under certain irradiation conditions. Hence, they resulted that these combinations did not have the potential as an alternative therapy (Ramírez et al. 2015). The discrepancies between our findings and those of the Ramírez et al. (Ramírez et al. 2015) study are likely due to differences in the PS type, dosages of light, therapy application order and strain‐specific characteristics. In this context, the clinical *A. baumannii* AB05 isolate used in the present study exhibited the weak biofilm‐forming ability. Since biofilm matrix components may act as a physical barrier that limits photosensitizer diffusion and reduces photodynamic efficacy, the relatively weak biofilm‐forming capacity of AB05 may have contributed to the enhanced susceptibility observed following NMB‐mediated aPDT and combination treatment (Fekrirad et al. 2021). Moreover, it may influence responsiveness to ROS‐mediated photodynamic damage and antibiotic‐aPDT interaction. Therefore, phenotypic variability among clinical *A. baumannii* isolates should be considered when interpreting the outcomes of combined aPDT‐antibiotic studies.

In agreement with the additive interactions observed in our study, several previous investigations have also reported the enhanced antibacterial activity of aPDT in combination with colistin against Gram‐negative pathogens (Pourhajibagher et al. 2017; Wozniak et al. 2019). Pourhajibagher et al. reported that toluidine blue O‐mediated PDT alone produced only partial antibacterial activity against pandrug‐resistant *A. baumannii*, resulting in less than 1‐log bacterial reduction. However, when combined with colistin, complete bacterial eradication with approximately 9‐log reduction was found at all tested colistin concentrations. In addition, the combined treatment reduced the MIC of colistin by more than 11‐fold (Pourhajibagher et al. 2017). Similarly, Wozniak et al. demonstrated synergistic interactions between rose bengal‐mediated aPDT and colistin against XDR *A. baumannii*, with FICI values of 0.375 for both tested isolates. In addition, CFU counting and post‐antibiotic effect analyses confirmed enhanced bacterial killing and delayed bacterial recovery following combined treatment (Wozniak et al. 2019). Overall, these findings suggest that colistin may potentiate the antibacterial efficacy of aPDT, possibly through membrane destabilization and enhanced photodynamic damage. Indeed, the membrane disruption induced by colistin may facilitate photosensitizer uptake and enhance susceptibility to ROS‐mediated photodynamic damage (Zhou et al. 2024; Richter et al. 2019).

In support of our findings with colistin, several studies using polymyxin B, a polymyxin‐class antibiotic with similar membrane‐disrupting activity, have also demonstrated enhanced antibacterial efficacy when combined with aPDT against Gram‐negative bacteria (Ucuncu et al. 2020; Le Guern et al. 2017; Richter et al. 2019). Le Guern et al. showed the growth inhibition of both Gram‐negative and Gram‐positive bacteria by a covalent methylene chlorin e6 (a PS) and polymyxin B conjugate (Le Guern et al. 2017). Also, Richter et al. showed that 10 mg/L chlorophyllin (a PS) alone could not inhibit the growth of *E. coli* or *Salmonella enterica* serovar Typhimurium. However, when chlorophyllin was combined with low dosages of colistin (0.50 or 0.25 µg/mL) under specific irradiation conditions, it completely eradicated the bacterial growth within the first hour (Richter et al. 2019). Ucuncu et al. in a research study first compared the efficacy of methylene blue and polymyxin B when they were co‐administered with that of a covalent methylene blue–polymyxin B conjugate. They confirmed that this conjugate had selective and potent aPDT activity against clinical isolates of *E. coli* and *P. aeruginosa* following irradiation, achieving 100% pathogen killing with short light irradiation times while showing no activity against *S. aureus*. Overall, these studies, together with our findings, suggest that polymyxin‐class antibiotics may potentiate the antibacterial efficacy of aPDT against Gram‐negative pathogens. However, the extent of these interactions (antibiotic and aPDT) appears to depend on the photosensitizer type, formulation strategy, irradiation conditions, and bacterial species.

The findings obtained from our study suggested that ROS generation increased following aPDT alone and combined antibiotic‐aPDT treatment, indicating that ROS may contribute to the enhanced antibacterial activity associated with the combined therapies. However, the present study did not include ROS quenching or mechanistic inhibition assays; therefore, the exact role of ROS in mediating these antibacterial effects remains to be fully clarified.

Similar to our findings, Wozniak et al. also reported increased ROS production following combined antibiotic/aPDT treatment against a clinical isolate of XDR *A. baumannii*, suggesting a possible role of oxidative stress in the enhanced bacterial killing (Wozniak et al. 2019).

While there have been numerous reports of successful outcomes, it is notable that the combination of PDT with antibiotics does not guarantee an enhancement in therapeutic efficacy in all cases. The nature and extent of the interaction between PDT and antibiotics seem to be influenced by several key factors, including the formulation of the combination therapy and the bacterial strain that is being subjected to treatment (Feng et al. 2021). Indeed, PDT is more effective against Gram‐positive bacteria rather than against Gram‐negative bacteria in regard to the different membrane structures and limited penetration ability of ROS (Naskar and Kim 2023).

## 5 Conclusion

This study confirmed the significant antibacterial activity of NMB‐mediated aPDT against a MDR *A. baumannii* strain in a concentration and fluence‐dependent manner. While NMB alone did not cause the full inhibition of *A. baumannii* growth, its combination with sublethal diode laser irradiation effectively reduced the viable count of this strain. In addition, synergistic and additive interactions were found in the combination of aPDT and sub‐MIC concentrations of tigecycline and colistin, which significantly rendered the enhanced eradication of this strain compared to monotherapies. ROS generation may represent one of the possible mechanisms contributing to the antibacterial efficacy of aPDT alone and combined antibiotic‐aPDT treatments. These findings underscore NMB‐mediated aPDT alone and in combination with colistin or tigecycline as a promising approach to eradicate infections associated with MDR *A. baumannii* that can potentially reduce the development of further antibiotic resistance.

Nevertheless, various experimental limitations should be noted during the analysis of the present results. The study design did not directly investigate the contribution of treatment order in the aPDT–antibiotic combination strategy, which may alter the observed synergistic efficacy. Moreover, the study did not completely distinguish between the photodynamic action and the possible influence of dark toxicity induced by NMB, particularly under higher NMB concentrations. Furthermore, comparisons between monotherapy and combination approaches of NMB and antibiotics were performed only at certain concentrations, which possibly reduced a complete evaluation of dose‐dependent interactions. As a result, our findings indicated the improved antibacterial activity of NMB‐mediated aPDT both alone and as part of combination therapy, but further investigation is essential to completely determine the specific role of each factor.

## Author Contributions

**Barat Barati:** investigation, validation. **Tahereh Navidifar:** writing – review and editing, writing – original draft, investigation, methodology. **Mohsen Ostovari:** methodology, formal analysis. **Roya Ghanavati:** formal analysis, writing – original draft. **Atieh Darbandi:** writing – original draft, investigation.

## Funding

The authors have nothing to report.

## Ethics Statement

The study protocol was approved by the Ethics Committee of Shoushtar Faculty of Medical Sciences, Shoushtar, Iran (approval number: IR. SHOUSHTAR.REC.1400.006).

## Conflicts of Interest

The authors declare no conflicts of interest.

## Data Availability

The data that support the findings of this study are available on request from the corresponding author. The data are not publicly available due to privacy or ethical restrictions.
